# Endoscopic Gold Fiducial Marker Placement into the Bladder Wall to Optimize Radiotherapy Targeting for Bladder-Preserving Management of Muscle-Invasive Bladder Cancer: Feasibility and Initial Outcomes

**DOI:** 10.1371/journal.pone.0089754

**Published:** 2014-03-03

**Authors:** Maurice M. Garcia, Alexander R. Gottschalk, Jonathan Brajtbord, Badrinath R. Konety, Maxwell V. Meng, Mack Roach, Peter R. Carroll

**Affiliations:** 1 Department of Urology, University of California San Francisco, San Francisco, California, United States of America; 2 Department of Radiation Oncology, University of California San Francisco, San Francisco, California, United States of America; 3 University of California San Francisco – Helen Diller Family Comprehensive Cancer Center, San Francisco, California, United States of America; 4 Department of Urology, University of Minnesota, Minneapolis, Minnesota, United States of America; University of Alabama at Birmingham, United States of America

## Abstract

**Background and Purpose:**

Bladder radiotherapy is a management option for carefully selected patients with muscle-invasive bladder cancer. However, the inability to visualize the tumor site during treatment and normal bladder movement limits targeting accuracy and increases collateral radiation. A means to accurately and reliably target the bladder during radiotherapy is needed.

**Materials and Methods:**

Eighteen consecutive patients with muscle-invasive bladder cancer (T1–T4) elected bladder-preserving treatment with maximal transurethral resection (TUR), radiation and concurrent chemotherapy. All underwent endoscopic placement of 24-K gold fiducial markers modified with micro-tines (70 [2.9×0.9 mm.]; 19 [2.1×0.7 mm.) into healthy submucosa 5-10 mm. from the resection margin, using custom-made coaxial needles. Marker migration was assessed for with intra-op bladder-filling cystogram and measurement of distance between markers. Set-up error and marker retention through completion of radiotherapy was confirmed by on-table portal imaging.

**Results:**

Between 1/2007 and 7/2012, a total of 89 markers (3–5 per tumor site) were placed into 18 patients of mean age 73.6 years. Two patients elected cystectomy *before* starting treatment; 16/18 completed chemo-radiotherapy. All (100%) markers were visible with *all* on-table (portal, cone-beam CT), fluoroscopy, plain-film, and CT-scan imaging. In two patients, 1 of 4 markers placed at the tumor site fell-out (voided) during the second half of radiotherapy. All other markers (80/82, 98%) were present through the end of radio-therapy. No intraoperative (e.g. uncontrolled bleeding, collateral injury) or post-operative complications (e.g. stone formation, urinary tract infection, post-TUR hematuria >48 hours) occurred. Use of micro-tined fiducial tumor-site markers afforded a 2 to 6-fold *reduction* in bladder-area targeted with high-dose radiation.

**Discussion:**

Placement of the micro-tined fiducial markers into the bladder was feasible and associated with excellent retention-rate and no complications. All markers were well-visualized during radiotherapy with all imaging modalities. Bladder fiducial markers improve targeting accuracy, and may increase treatment efficacy and reduce morbidity from collateral radiation.

## Introduction

Radical cystectomy with urinary diversion remains the gold-standard treatment for localized muscle-invasive bladder cancer. However, bladder preservation with tri-modality therapy (maximal transurethral resection, and combined chemo and radiotherapy) remains an alternative for patients who have been thoroughly counseled regarding the attendant risks and benefits of all management options, including radical cystectomy [1]. Ideal candidates for bladder sparing management should have low stage, focal disease that is amenable to complete transurethral tumor resection [2]. Despite the absence of direct randomized trials comparing bladder preserving treatment and radical cystectomy, tri-modality treatment with maximal transurethral bladder tumor resection followed by different regimens of combined radio and chemotherapy has, among patients with the aforementioned disease features, achieved results comparable to radical cystectomy in some trials [3].

Two key challenges to radiotherapy targeting accuracy and precision are that the location of the bladder moves constantly, and, that the tumor-site cannot be identified using CT-scan imaging or on-table imaging. Consequently, the location of the bladder tumor site during treatment cannot be predicted by the planning-CT.

The need to improve the radio-therapeutic window is *essential* to improve clinical outcomes using radiotherapy for bladder cancer. The inability to localize the bladder walls at time of daily treatment using current “on table” imaging modalities (e.g. portal and cone-beam CT), results in: 1. *Under-treatment* because the entire target (bladder), or a portion (bladder tumor site), are not treated consistently throughout the (generally) 4–6 weeks of radiotherapy, and 2. *Over-treatment*: delivery of radiation to collateral non-bladder tissues, or, delivery of high-dose radiation to the bladder outside of the tumor site target area.

Twenty-Four karat gold prostate fiducial markers (placed into the apex and base of the prostate gland) have been shown to significantly improve targeting and decrease collateral radiation during radiotherapy for prostate cancer [4]. [5] The high isodenisty of pure gold offers excellent visualization with portal imaging. Given the inability to adequately target the tumor site for bladder radiotherapy for muscle-invasive bladder cancer, we sought to design fiducial markers and a placement protocol for use to define the location of bladder tumor. Because prostate fiducial markers have been shown to migrate [6] (decreases accuracy and increases collateral radiation), we manufactured our markers with micro-tines along their sides, to anchor each into place within the submucosa. We sought to optimize our marker delivery protocol to minimize risk for fall-out and migration. We also sought to test whether the marker's size (and thereby delivery-needle diameter/gauge) could be reduced.

Beginning in January 2007, we designed a fiducial marker and placement protocol to mark the tumor site in 15 consecutive patients with localized muscle invasive (≥T1NxM0) transitional cell bladder cancer. Patients were thoroughly counseled regarding the risks and benefits of all management options, including the gold-standard– radical cystectomy and urinary diversion, in addition to bladder preserving multi-modality treatment. All markers were placed endoscopically into intact bladder submucosa surrounding the tumor resection site.

The present work is a feasibility study, and describes our fiducial marker design, placement technique, and outcomes of our early experience.

## Materials and Methods

### Ethics statement

All patients gave written informed consent to undergo treatment and participate in this study, and approval for this study was obtained from our institution's (University of California San Francisco) Committee for Human Research.

Between January 2007 and July 2012, a total of 18 consecutive patients with focal, T2, NX, M0 urothelial carcinoma, elected bladder-preserving multi-modality treatment and underwent endoscopic placement of fiducial markers into the bladder wall (13 men and 5 women, mean age 73.7 and 73.4 years, respectively). A total of 89 fiducial markers were placed into healthy bladder submucosa ∼0.5 cm. from the resection margin.

### Fiducial markers and placement technique

**Figure 1:**
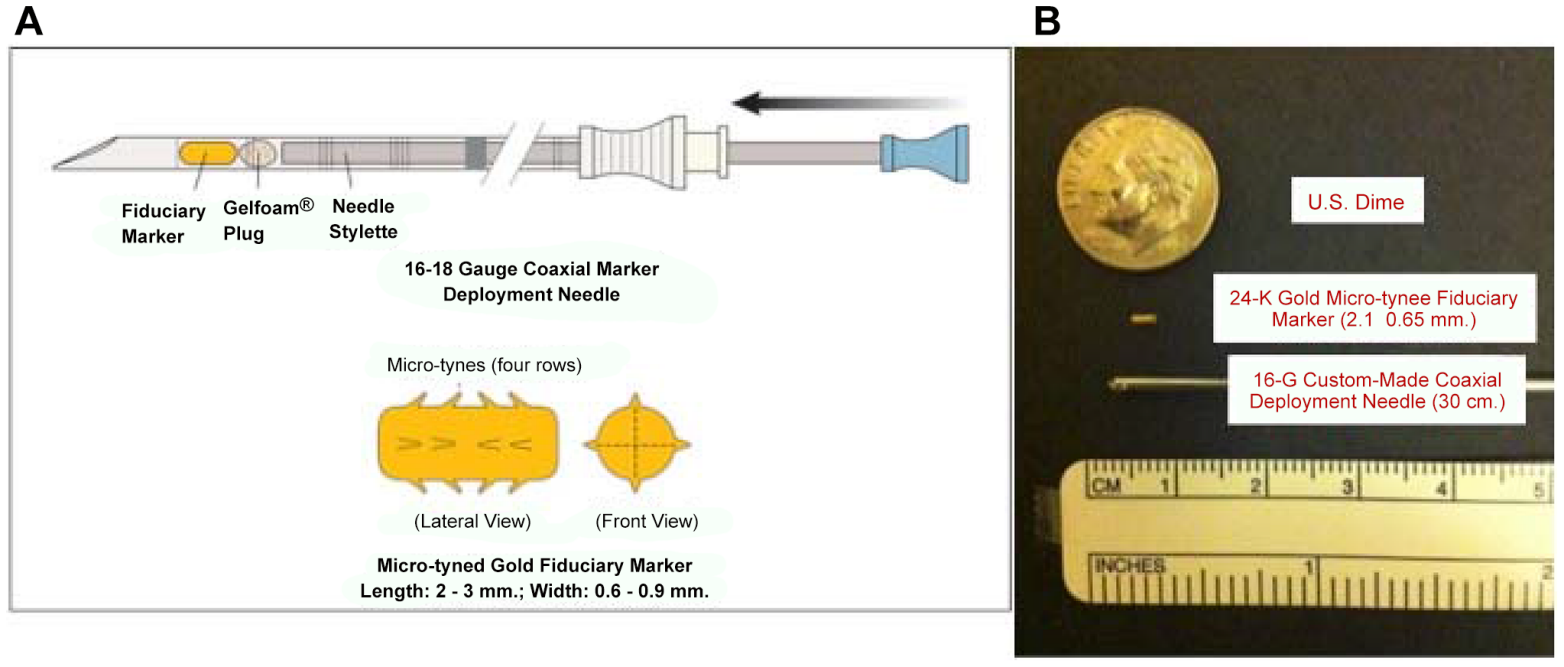
Gold bladder fiducial markers, Gelfoam™ plug, and custom-made coaxial deployment needles. **A.** Each 24-K gold micro-tined fiducial marker is manufactured with four length-wise rows of tines, whose points all face toward the center of the marker. The purpose of the micro-tines is to prevent the marker from migrating within the submucosal space. Each marker is deployed via a custom-made 30-cm. coaxial 18G needle into bladder submucosa. To prevent the marker from falling-out via the hole created by the placement needle, a small “plug” made of morcellated Gelfoam™ is deployed with (behind) the marker. **B.** 2.1×0.65 mm. gold fiducial markers are shown beside a custom-made 30-cm. coaxial 18G needle, and a U.S. dime.

**Figure 2:**
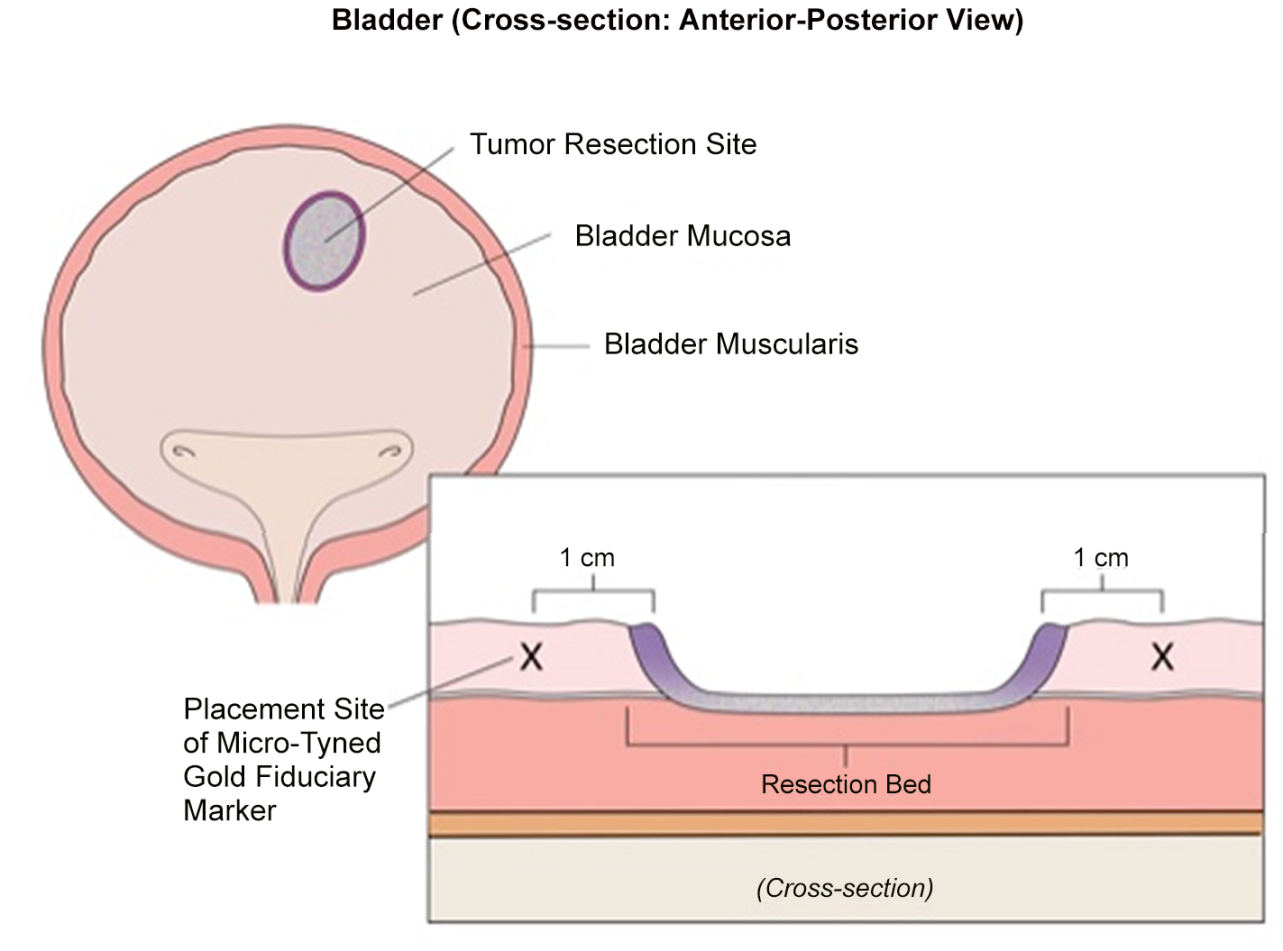
The tumor resection site is marked by a minimum of three fiducial markers placed circumferentially. Electrocautery will cause necrosis of mucosa immediately adjacent to the resection site (*purple*). For this reason, we place the fiducial markers into healthy mucosa ∼5–10 mm lateral to the resection margin (“X”).

**Figure 3:**
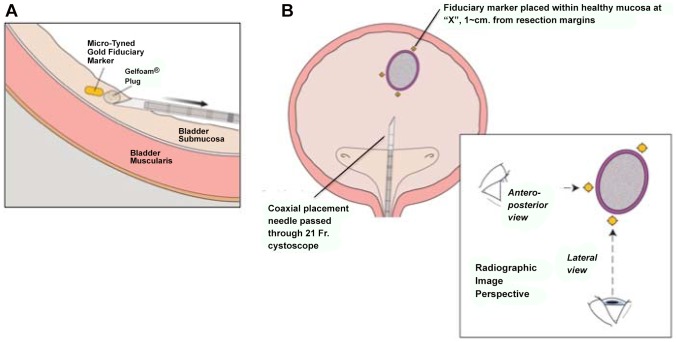
**A.**During placement, the tip of the deployment needle should be tunneled (approximately parallel to the bladder wall) a short distance beneath the mucosa before the stylet is advanced to deploy the marker and Gelfoam™ plug. Submucosal tissue collapses upon the micro-tines, to anchor the fiducial marker in place and prevent marker migration. **B.** A *minimum* of three markers are placed around a single tumor site. Markers should be oriented circumferentially in a trident (e.g. Mercedes®-sign) rotated 30–60-degrees, such that when the three+ markers are viewed by radiograph from either an anterior-posterior view and a lateral view, each is distinct and does not overlap with the others.

All fiducial markers were 24-K gold (W.E. Mowrey, St. Paul, MN.). Each was modified in our laboratory to have four lengthwise rows of *micro-tines*, and was re-sterilized before placement. (**Figure 1**) Each marker was delivered into healthy bladder-submucosa (∼5 mm from the tumor resection margin; **Figure 2**) using custom-made 30 cm. 18G and 16G coaxial needles (Popper & Sons, NY, USA) (**Figure 1**). A “plug” of morcellized Gelfoam™ was delivered with (behind) each marker, so that it resided between the marker and the hole into the submucosa created by the deployment needle. (**Figure 3A**). Care was taken to advance the needle-tip obliquely beneath the bladder mucosa only the *minimal* distance necessary to bury the open needle-end within submucosa, before deploying the marker. A total of 3 to 5 micro-tined fiducial markers were placed circumferentially around each tumor resection site in a staggered orientation, such that from all markers were visible from *both anterior-posterior* and *lateral* radiographic views (**Figure 3B**). A single surgeon (MMG) placed all of the markers. Diathermy was not routinely used to control bleeding at the marker placement sites.

### Fiducial marker placement at the bladder anatomic margins

For the most recent 5 patients in our series, following marker placement at the tumor resection site, fiducial markers were also placed at the anatomic boundaries of the bladder (center posterior wall, center of the left and right lateral-most point of the lateral wall, and center dome), under combined direct and fluoroscopic guidance.

Technique: A 16 Fr. urethral catheter was placed per urethra. The bladder was filled with 60 ml. of *diluted* contrast/saline solution, to allow visualization of the bladder lumen margins, a cystoscope, delivery needle, and fiducial markers, under fluoroscopy. Care was taken to ensure that the C-arm fluoroscope remained fixed in place relative to the patient at all times. The cystoscope was then inserted into the bladder, and *under fluoroscopic guidance*, the tip was positioned at the desired anatomic margins (lateral-most side wall, dome center, center posterior wall). A single fiducial marker was deployed at each site under fluoroscopy.

**Figure 4:**
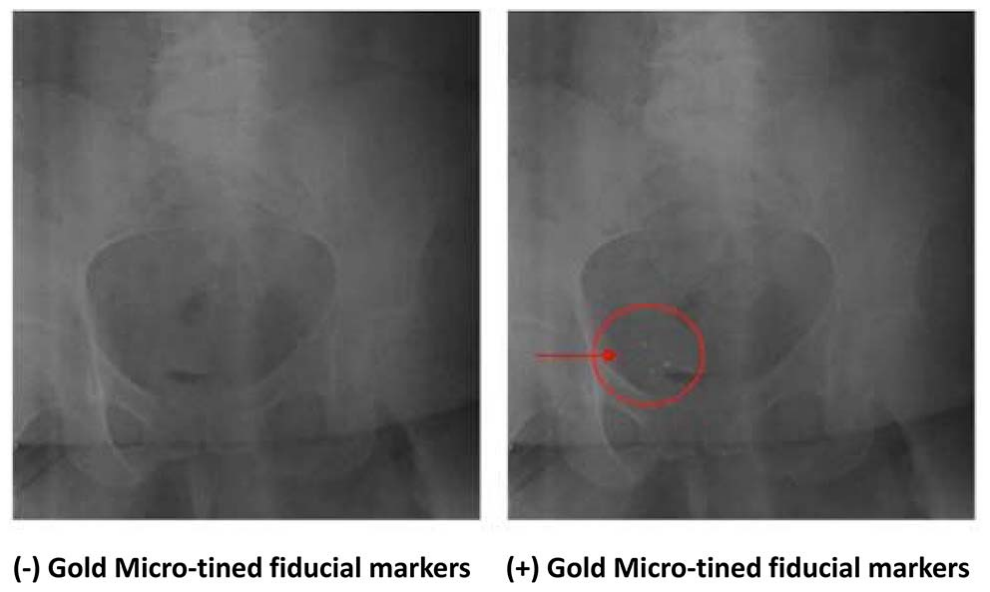
Plain-film X-Ray images of the pelvis *without* (left) and *with* (right) three 2.1×0.65 mm. 24K gold micro-tined fiducial markers surrounding the resection site of a right posterior-lateral bladder wall tumor.

For the first 12/18 patients in our series, all underwent intra-operative Fluoroscopy and post-operative pelvis pelvic X-Ray imaging to confirm placement and location of all markers. (**Figure 4**)

### Assessment for intraoperative migration using filling cystogram and a smartphone App

**Figure 5:**
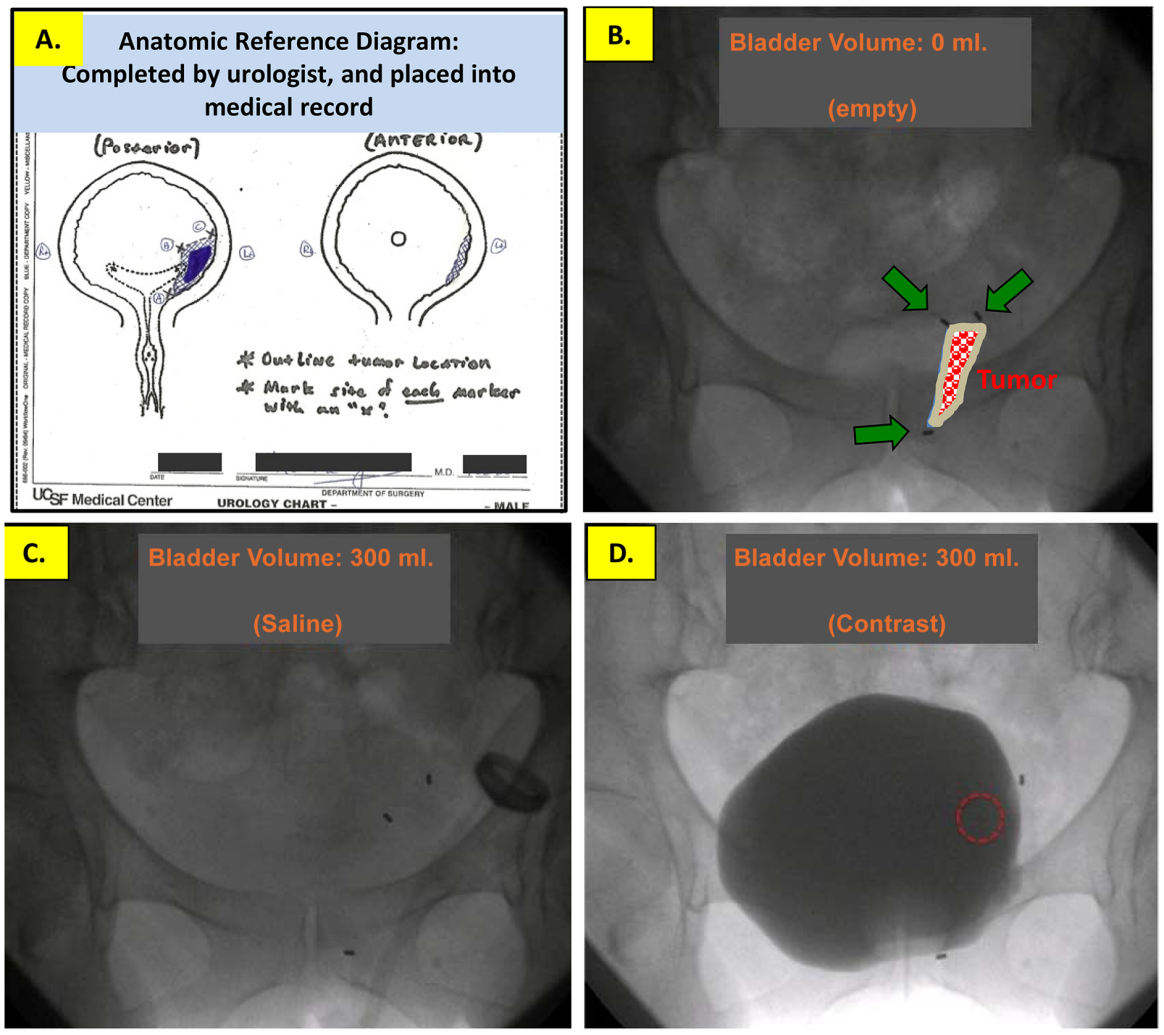
Intraoperatively, we completed a *bladder map* pictogram of the anterior and posterior walls of the bladder. On this bladder map we drew the location of the bladder tumor, additional biopsy sites, and, abnormal anatomy (e.g. diverticulae, ectopic ureteral orifice, etc.), and, the location of each fiducial marker placed (**A.**). We numbered each marker on the bladder map, and also referred to the numbered markers in the dictated operative note. The bladder map was entered into each patient's electronic medical record, for future reference by our Radiation Oncology colleagues during dosimetry planning. Intraoperative fluoroscopic images of the catheterized bladder following placement of three fiduciary markers at the site of a left anterior-lateral bladder wall tumor: (**B.**) Bladder empty. The location of the tumor and resection margin are outlined by three fiducial markers; center is filled in color. The bladder was then filled with 300 cc. of saline (**C.**), and separately with diluted contrast (**D.**), to assess marker movement with bladder filling and to compare the location of each marker during independent filling studies of equal volume.

In the last 6 patients, to confirm that the markers tracked the bladder wall throughout filling/emptying and did not migrate intraoperatively, we also performed an intra-operative contrast filling study under C-arm fluoroscopy. The C-arm was fixed in place over the patient's pelvis and the bladder was imaged empty and then during filling and emptying (60 ml increments, from 0 to 240 ml., then 240 to 0 ml.). A spot fluoroscopic image was taken after each 60 ml. change (+/−) in volume. This was performed first with saline, followed by a repeat series using diluted contrast (**Figure 5**). As both the patient's pelvis and the C-arm remained fixed in 3-dimensions throughout filling/emptying, any change in the location of the fiducial markers could be detected and measured. We now routinely fill the bladder with only 240 ml. (saline and diluted contrast), as this lower volume is sufficient to demonstrate marker tracking of the bladder wall.

**Figure 6:**
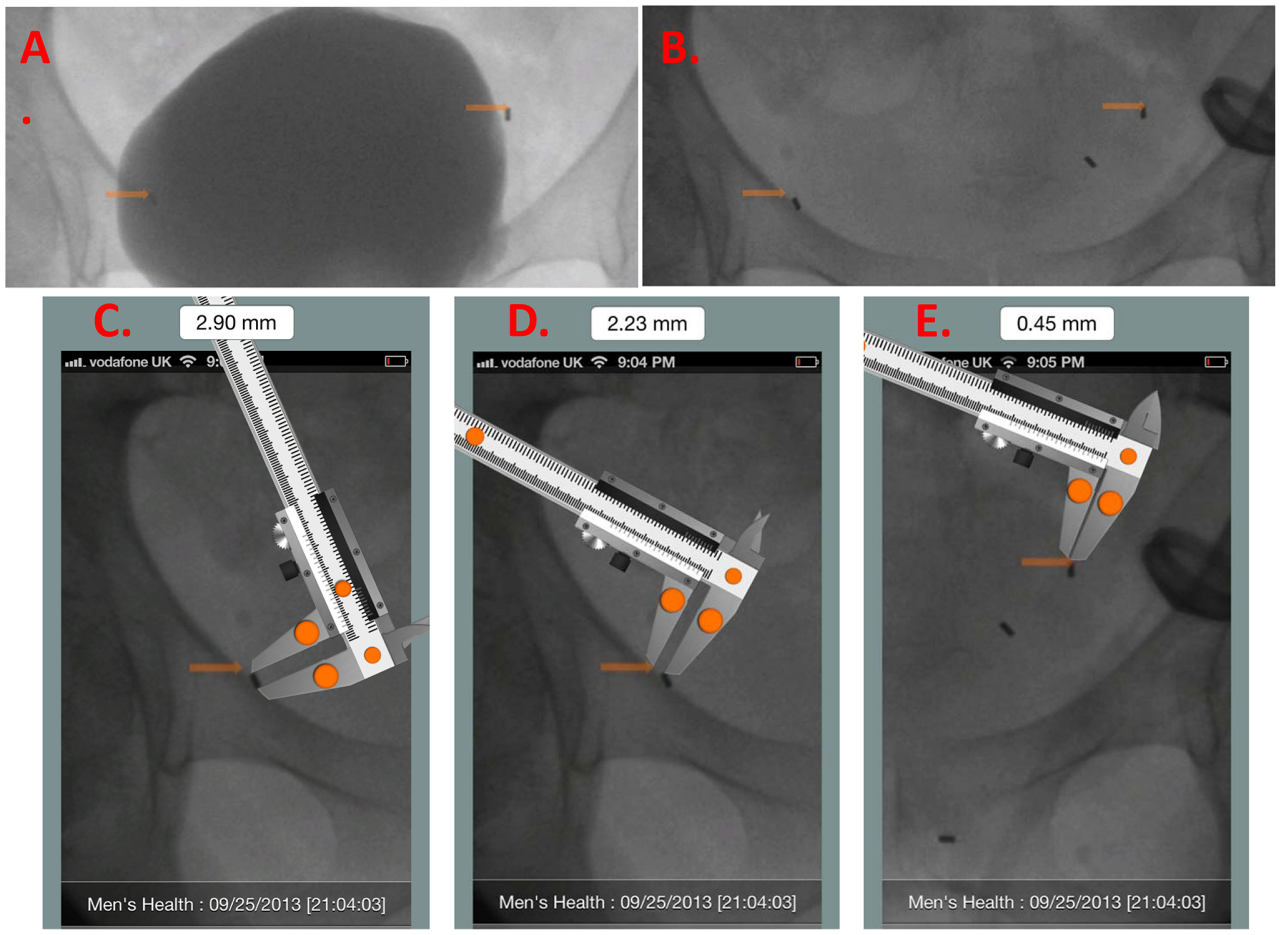
In order to confirm that the fiducial markers move apart with bladder filling *and* move together with bladder emptying, motion, immediately after all markers were placed, we performed a volumetric filling/emptying cystogram. Using diluted contrast, the bladder was serially filled 0-arm remained fixed in position. The same procedure was repeated, separately, with saline. At each incremental 60 ml. change in bladder volume, we obtained a spot-fluoro image. We compared images of a patient's bladder filled with equal volumes of dilute contrast (**A**) and saline (**B**). We then used the digital image measurement App (MedMeasure!; U.S. and International Patents Pending) [https://itunes.apple.com/us/app/medmeasure!/id654898049?mt=8] to measure the distance between pairs of markers in paired images of the bladder filled with the same volume of saline or contrast. The digital, scalable caliper provided by the MedMeasure! App is first calibrated to one of the fiducial markers visible in lateral view– whose length of which is known to equal 2.9 mm. (**C**). Upon calibration, the *actual* distance between any two markers can be measured with the caliper. We measured the *difference* in distance between each marker-pair in paired images. (**D, E**).

For each bladder filing volume (i.e. 0→60→120→180→240→240→180→120→60→0 ml.), we compared the location of each marker in the contrast-filled versus saline-filled bladder image (**Figure 6 A, B**).

We then used an image-based anatomic measurement smartphone App that we (MMG) designed (MedMeasure!, U.S. and International *Patents Pending*, https://itunes.apple.com/us/app/medmeasure!/id654898049?mt=8) to measure the distance (mm.) between matched pairs of fiducual markers at any given equal volume of saline vs. contrast.

Technique: A photograph of the fluoroscopy image showing the fiducial markers within the bladder was uploaded into the App. A digital caliper was superimposed on the image within the App. The caliper was calibrated to a known actual length (the actual length of a lateral view of one of the fiducial markers; = 2.9 mm.). (**Figure 6C**). Once calibrated, the caliper was re-positioned on the image to measure actual (i.e. *real*) distance between matched marker pairs. (**Figure 6D, E**)

### Bladder tumor and fiducial marker mapping; post-op management

We used a schematic bladder-map of the bladder to indicate (intraoperatively) the tumor resection location in 3-dimensions, and, the location of all fiducial markers (**see** **Figure 5** **& Video S1**). We also annotated any anatomic abnormalities (e.g. bladder diverticulum, previous resection scars, ectopic ureteral orifice). Each fiducial marker was numbered on this map and was referenced in the operative report. The “bladder map” was then placed into each patient's electronic medical record, for easy reference by our Radiation Oncology colleagues. All patients received an oral prophylactic antibiotic for 5 days post-operatively. All patients were contacted by telephone 24, 48, and 72 hours post-operatively for follow-up assessment.

**Figure 7:**
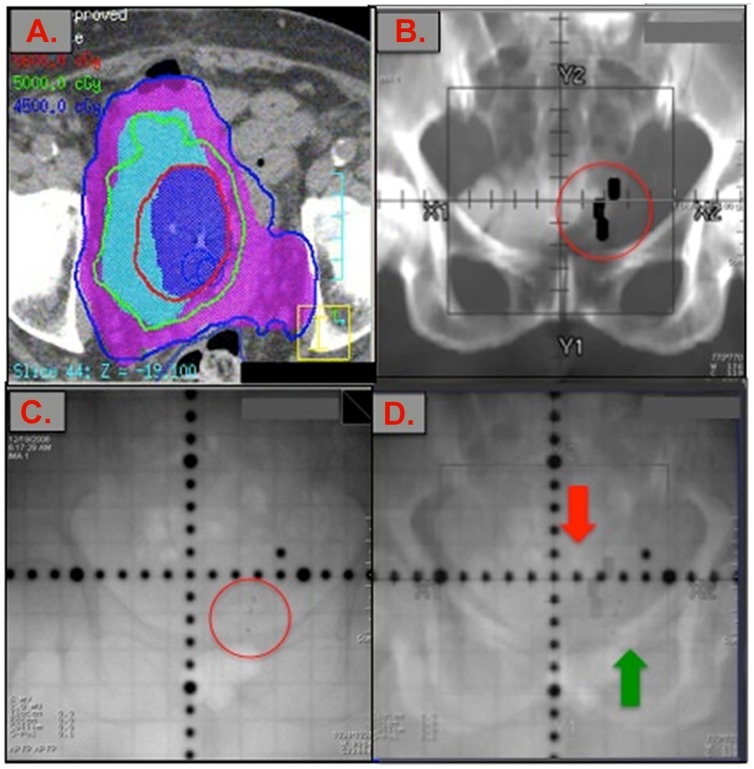
Computed Tomography (CT), Digitally Reconstructed Radiograph (DRR), and on-table Portal images with fiducial markers present. (**A.**) Computed Tomography (CT-scan) dosimetry imaging of pelvis with bladder fiducial markers in place. The gross tumor volume (GTV; dark blue) received 56-Gy in 25 fractions. The clinical tumor volume (CTV; light blue) equals the bladder and received 50 Gy in 25 fractions. The planning tumor volume (PTV; purple) equals the CTV+1 cm + the ipsilateral pelvic side-wall. (**B.**) Digitally reconstructed radiograph (DRR) of pelvis; shows the *anticipated* location of the three markers we placed at the tumor site (large black rectangles), based on the gold markers visible on planning CT. (**C.**) Gold markers seen on an AP portal image of pelvis, (*circled*), (**D**) Fusion of the DRR and AP portal images showing the predicted position for gold markers (red arrow) compared to the true position of the gold markers (green arrow) prior to any patient adjustment.

All patients who proceeded to radiotherapy underwent pre-treatment computed tomography (CT) scan imaging for dosimetry planning and portal imaging at time of daily radiotherapy (**Figure 7**).

### Use of markers to assess potential on-table set-up/targeting error during radiotherapy treatment visits

At treatment, patients were aligned ‘on-table’ with one of two techniques: 1. Daily anterior-posterior (AP) and lateral-view portal images were taken and the patient's gold markers were aligned to the gold markers on the reference digitally recreated radiograph, or, 2. Daily MV cone-beam CT was performed and the patient was aligned to the gold markers on the planning CT images.

To assess whether the presence of the markers improved targeting, for the last 5 patients in our series, we measured set-up error on the first 3 days of treatment as follows: patients were aligned daily to skin marks using a 3-point set up. Portal images were taken and overlaid with the planning Digitally Reconstructed Radiograph (DRR). The position of the gold fiducial markers on the planning CT and the daily on-table portal image were compared. All patients were treated with IMRT using 6 MV photons.

## Results

A total of 89 markers were placed into 18 patients (**Table 1**). A minimum of 3 to 4 “regular” size markers were used to mark the tumor site. In the last 9/18 patients, a total of 21 “extra-small” (2.1×0.65 mm.) markers were also placed, each usually in combination with a “regular” size (2.9×0.9 mm.) marker. Occasionally more than 3 (up to 5) markers were placed at the tumor site if it was felt that one or more of the first 3 markers was at risk of falling-out prematurely- usually due to deployment too close to the mucosa needle-entry hole.

**Table 1:** Summary of Patient Clinical Data.

N = 89 markers/18 patients								
Sex	Age (yrs)	Stage	# Markers placed	Started Chemotherapy & Radiation	Completed Chemotherapy & Radiation	No. markers present through end of radiotherapy	Intra-Op Complications	Post-Op Complications
Men: 13	73.7	T1 (3)	89	16/18	16/16	80/82 (98%)	0/18 patients	0/18 patients
Women: 5	73.4	T2 (11)			(100%)			
		T3 (2)						
		T4 (2)						
							**Examples:**	**Examples:**
Total: 18			2.9×0.9 mm. = 68	(2 opted for cystectomy			Prolonged	UTI; abscess;
			2.1×0.65 mm. = 21	*before* starting			bleeding;	Prolonged
				chemo-radiotherapy)			Injury to	bleeding;
							collateral	Prolonged pain;
							structures	Stone formation

In the last 5 patients in this series, 2–4 fiducial markers were also placed at one or more bladder anatomic boundaries (center of lateral-most side walls, posterior wall and dome). In all (18/18) patients, all markers (82/82; 100%) were clearly visible on intra-operative fluoroscopy, and post-op pelvic X-ray (**Figure 4**). Among patients who started radiotherapy, all (100%) markers were visible on CT scan, and MV/cone-beam portal imaging (**Figure 7**).

No (0%) intraoperative or post-operative complications occurred. This includes hematuria beyond what can be expected flooring transurethral tumor resection (>48 hours, hematuria requiring transfusion or clot evacuation), UTI, injury to collateral pelvic structures, development of bladder calculi, or chronic pain. In our series, we used electrocautery to control mucosal bleeding only once. We limited its use to avoid tissue necrosis and delayed sloughing at the marker site – which would result in delayed marker loss. In our experience, bleeding at the marker site is generally minimal, and resolved spontaneously with the aid of a urinary catheter left in place post-op and removed prior to same-day discharge from hospital. Hematuria resolved within 24 hours for the majority of patients, and within 48 hours post-operatively for the remainder. At follow-up cystosopy, no patient demonstrated bladder calculi. For each of two patients, one marker fell-out *during placement* (as a result of the placement needle not being advanced sufficiently into the sub-mucosal space). In both instances, the marker was retrieved with an endoscopic grasping instrument, and immediately re-placed. To ensure that the open portion of the beveled needle-tip is completely submucosal, we found it most helpful to withdraw the needle very slightly- simply to allow visualization of muscoa around the needle tip, before advancing the stylet.

Two of 18 patients elected to undergo cystectomy before initiating chemo-radiotherapy. The remaining 16 patients (82 markers) completed chemo-radiation therapy. In two patients early in our series, two of the 4 markers placed fell out during the last quarter of radiation therapy, resulting in a net marker-loss rate of 2/82 markers = 2.4%. All other markers (80/82, 98%) were visible and remained in place through the end of radiation therapy.

### Assessment for intraoperative migration using filling cystogram and a smartphone App

In all 5 patients evaluated, the markers appeared, grossly, to remain in place during and after bladder filling with saline versus diluted contrast. (**See Video S1**) The MedMeasure! App © was used to calculate the mean change in location of each marker on fluoroscopic images taken during bladder filling with equal volumes of saline versus contrast, which was 2.32 mm. (range: 0.10–9.7 mm. (**Figure 6**) All 5 patients had *at least one* matched-volume inter-marker distance measurement difference that was <1 mm. and which occurred during the emptying phase of the serial cystogram.

### Use of markers to assess potential on-table set-up/targeting error during radiotherapy treatment visits

During treatment of 5 patients, on each day of treatment, the patient was initially aligned upon the table using only conventional methods (skin tattoos, bony landmarks). Then, without the aid of on-table spot-imaging, the Radiation Oncologist attempted to predict the location of each of the gold fiducial markers within the patient's pelvis by consulting the archived *dosimetry-planning* non-contrast CT scan images, which showed the fiducial markers. The Radiation Oncologist then super-imposed dark-black rectangle shapes upon the pelvis of the patient on the table – to mark where he/she expected each marker to be (**Figure 7, B**). Next, on-table imaging was performed which showed the actual location of the markers (**Figure 7, C**). A Digitally Reconstructed Radiograph (DRR) was made which showed the markers actual location within the patient on-table, and their predicted location based on the dosimetry-planning CT (black-shapes, **Figure 7, D**). The distance between the expected vs. actual location of the fiducial markers was measured in the X and Y dimensions. The discrepancy between predicted versus actual location of the markers was classified as “set up error”. The average set up error in the X-dimension was 1.96 cm. (range: 0.76–2.14), and in the Y-dimension it was 0.95 cm. (range: 0.52–1.74)

### Post-radiotherapy cystoscopy

All patients underwent cystoscopy approximately midway through radiotherapy treatment and then every 3–6 months following treatment. In all patients, the bladder mucosa covered the fiducial markers (i.e. markers were not visible), and no patient (0%) demonstrated bladder calculi.

## Discussion

The bladder wall is a mobile structure and is not well-visualized using on-table radiographic imaging available during radiotherapy. An inability to visualize the exact location of the tumor resection site precludes targeting it with high-dose radiation during daily radiotherapy. We have shown that the novel gold fiducial markers we describe can be easily delivered endoscopically into bladder submucosa and appear to remain in place to identify the tumor site and bladder-margins throughout radiotherapy. Our intra-operative volumetric cystogram under fluoroscopy that the distance between a matched-pair of markers varies generally little (mean 2.32 mm.) but can vary up to ∼1 cm. Because all patients had at least 1 inter-marker distance measurement (after the maximum discrepancy was detected) that was <1 mm., it is unlikely that the markers were actually migrating, but rather, that the variance in inter-marker distance reflects changing position of the bladder wall at the marker site(s).

In our experience, our markers improve on-table alignment accuracy and precision. On table imaging throughout radiotherapy showed that >98% of these fiducial markers are retained and remain in place relative to one another and did not migrate. Follow-up evaluations, including cystoscopy, confirm that the patients in this series had no adverse sequelae that could be attributed to the markers, such as bother, stone formation, or infections.

Modern radiation-therapy techniques permit dose escalation to the target without increasing the dose to the surrounding normal tissues [7], [8]. However, the location of the bladder on dosimetry-planning CT-scan cannot accurately predict the exact location of the bladder at treatment. The location of the bladder walls changes constantly (changing urine volume, bowel activity, and rectal distention [9], [10]). Also, the tumor site cannot be identified with CT-scan imaging, As a result, the field area (tumor site) targeted for high-dose radiotherapy (HDR) (aka. *Gross Tumor Volume*, GTV) during dosimetry planning is over-sized, which increases collateral radiation with HDR [10]–[14]. Because the volume of a *sphere* is equal to 4/3π r^3^ ([15]), any increase in the radius of the target area results in a large increase in the volume of irradiated field. For example, conformal radiation of an actual 2 cm-wide tumor results in a HDR volume of 33.5 cm^3^, whereas if the field area is increased to a 4 cm width, the irradiated HDR volume is 268.1 cm^3^. Collateral radiation increases the incidence of treatment morbidity: bladder/pelvic tissue ulceration, bleeding, urinary frequency, urgency, and stone formation. [16], [17]

Past efforts to account for bladder wall motion have not been reliably successful. Use of external bony landmarks and skin tattoos simply cannot improve targeting of the bladder wall. For example, in our work, the mean discrepancy noted between the location of the fiducial markers as visualized with on-table imaging versus their predicted location based on the planning CT scan DRR superimposed onto the on-table image was 1.96 cm. in the X-dimension and 0.96 cm. in the Y-dimension. This measured discrepancy reflects the fact that bladder motion can contribute significantly to under-targeting of the tumor site and to collateral radiation. It also suggests that the calculated set-up error likely underestimates common set-up error to target the tumor site because in this small experiment, the radiation oncologist was allowed to use the location of the fiducial markers on the dosimetry-planning CT to estimate their actual location within the patient at moment of treatment. Without this aid, set-up error could be greater.

Strategies to control bladder volume during radiotherapy (e.g. drinking protocols [7], [8], [12], [14]) and treatment verification protocols using clips [18] have not been successful. Sondergaard et al. report use of Lipiodol solution injected around the tumor site [19]. This technique has several limitations, which include dependence on cone-beam CT imaging for visualization during intra-operative injection at time of treatment, and, risk of solution migration within or through the bladder (which when un-noticed, results in over and/or under treatment). Ideally, the area of any fiducial marker should not confound the targeting of the area it is supposed to define. Hence, fiducial markers should ideally be ‘pin-point’ in nature. Lastly, to be useful, markers must remain in place throughout radiotherapy.

At our institution, owing to the uncertainty of tumor location using on-table imaging and to anticipated bladder wall movement, in order to minimize the risk of under-treating the tumor site with HDR, approximately ∼¼ of the total bladder area is targeted with HDR. Because our fiducial markers allow us to identify the tumor site *independent of bladder motion* using standard on-table imaging, we were able to reduce the area of bladder targeted for high-dose radiation by a factor of *2 to 6-fold* .

We devised and began using our fiducial marker design, devices and approach this before any published reports describing the use of gold fiducial markers in the bladder. Mangar et al. published the first report describing the use of gold fiducial markers placed endoscopically into the bladder wall [20]. However, they did not report any assessment to confirm that their markers actually tracked the bladder wall during filling and emptying, and 25% of their markers were lost before completion of radiotherapy. Our marker design and approach is different in key respects. Ours have a micro-tined surface and are significantly shorter in length (2–3 mm vs. 10 mm used by Mangar). Our experience using the smaller of the two fiducial marker sizes we used confirms that markers as long as small as 2.1 mm by 0.65 mm can be seen in plain radiograph films, dosimetry-planning CT, and during treatment, on cone-beam CT and portal imaging. Mangar et al. used gold fiducial markers that were significantly longer (8 and 10 mm.), which possibly contributed to their high immediate marker fall-out rate (1 in 4).

Furthermore, in contrast to the flexible placement needle used by Mangar et al, we used a rigid coaxial needle/stylet, which affords complete control of the needle-tip depth of penetration. To prevent immediate marker fall-out via the needle entry-hole in the submucosa, we deliver a small amount of morcellized *Gelfoam™* behind each marker. To further prevent marker fall-out and migration, we *tunnel* our placement needle under the mucosa before deploying the marker with the stylet, thus creating a “flap” of tissue which coapts around the marker and, with the help of the micro-tines, helps to secure it in place. We strictly refrain from routine use of diathermy at the placement site because the resulting tissue necrosis will likely result in delayed marker fall-out. We place markers *only* at the site of healthy/intact submucosa, ∼5 mm. from the resection edge because tissue closer to the resection-bed edge is will necrose and result in marker fall-out. We use of custom-made *long* coaxial delivery needles because cystoscopes are too long for use of long spinal needles or prostate fiducial marker-placement needles.

A potential limitation of multi-modality therapy is that, ideally, it should be practiced with close communication between the urologist performing tumor resection/marker placement and the treating radiation oncologist(s). Our fiducial marker protocol speaks to this need: the intraoperative bladder-map informs the radiation oncologist(s) about what was seen intraoperatively (anatomic irregularities: e.g. bladder diverticulae, satellite and/or irregularly shaped tumors), what tumor border each marker identifies, and, the number and location of all markers. Also, if a marker subsequently falls-out, it can be readily identified with this bladder map. The images from the dilute-contrast filling study we now routinely perform are regularly used by our radiation oncology colleagues during dosimetry planning.

The present work serves principally to demonstrate the feasibility and safety of our fiducial marker placement technique. Future studies, with a greater number of patients and combined with inter-fraction plain imaging, are needed to more rigorously exclude marker migration. Future studies are also needed to assess clinical outcomes among patients treated with bladder fiducial markers (a report by our group on this is in progress). To formally assess benefit regarding targeting accuracy and collateral radiation, future studies should include an extra dosimetry-planning CT scan and *Dose Volume Histogram* (DVH), made *before* marker placement (i.e. blinded), so that this can be compared to the dosimetry-planning CT *after* marker placement. Future work should also explore the potential role of fiducial markers in combination with newer treatment technologies, such as, for example, Cyber Knife. Lastly, future work should report on clinical outcomes among patients treated with fiducial-marker targeted radiotherapy (work underway by our group), to potentially further refine placement protocols and device design.

## Supporting Information

Video S1 **Three markers were placed to mark the tumor site of a patient with unifocal T2 disease.** Immediately after placement, under Fluoroscopy, the empty bladder was filled and then emptied (via a urethral catheter) with dilute contrast in serial increments of 60 ml. (filling: 0 ml. to 240 ml, followed by emptying: 240 ml to 0 ml.) A Fluoroscopic image of bladder was recorded at each volume. So that the location of all markers could be easily compared relative to one another and to bony landmarks, the position of the Fluoroscopy unit and patient were fixed in space throughout imaging, so that the only source of movement was the patient's bladder and GI motility. Comparisons of images of the fiducial markers within the bladder while the latter was filled with an equal volume of either saline versus diluted contrast confirms that the markers move *with* the bladder-wall as the bladder expands and contracts during filling and emptying. Also, rapid serial images of the bladder were captured while holding intravesical volume constant. These images show how both the abdominal wall, intestines and the bladder-wall move in 3D with visceral peristalsis and inspiration.
(MOV)
