# Correction: Endoscopic Gold Fiducial Marker Placement into the Bladder Wall to Optimize Radiotherapy Targeting for Bladder-Preserving Management of Muscle-Invasive Bladder Cancer: Feasibility and Initial Outcomes

**DOI:** 10.1371/journal.pone.0164558

**Published:** 2016-10-06

**Authors:** Maurice M. Garcia, Alexander R. Gottschalk, Jonathan Brajtbord, Badrinath R. Konety, Maxwell V. Meng, Mack Roach, Peter R. Carroll

There is an error regarding the consent procedure reported within the Ethics Statement of the Materials and Methods section. The written informed consent obtained was for clinical treatment with markers, and not part of a prospective research study as is suggested by the statement. The Ethics Statement should read: Approval for this study was obtained from our institution's (University of California San Francisco) Committee for Human Research. Written informed consent was only obtained for clinical treatment with the study’s markers.
